# SOCIAL RESOURCES PREDICT EMOTIONAL WELL-BEING IN COGNITIVELY IMPAIRED OLDER ADULTS: RESULTS FROM THE ARMADA STUDY

**DOI:** 10.1093/geroni/igae098.0474

**Published:** 2024-12-31

**Authors:** Melanie Johnson, Claire Growney, David Salmon

**Affiliations:** Shiley Marcos Alzheimer’s Disease Research Center, University of California, San Diego, San Diego, California, United States; Stanford University, Stanford, California, United States; University of California San Diego, La Jolla, California, United States

## Abstract

As older adults tend to prioritize meaningful relationships, they are likely to have higher quality social relationships, which may contribute to maintained or improved emotional well-being with age. Social resources may be especially important for maintaining well-being among older adults with neurodegenerative diseases. We utilized NIH Toolbox (NIHTB) measures to examine group differences in the associations between social resource quality and emotional well-being among an ethnoracially and cognitively diverse sample of older adults. As part of the Assessing Reliable Measurement in Alzheimer’s Disease and Cognitive Aging (ARMADA) study, older adults who were cognitively normal (CN-Young: aged 65-84; n=161; CN-Old: aged 85+; n=116), with amnestic mild cognitive impairment (MCI; aged 64+; n=109), and with Alzheimer’s disease (AD; aged 62+; n=76) completed NIHTB measures of emotional well-being and social resource quality. Results from linear regression models indicated that social resource quality was associated with better emotional well-being on all thirteen emotional well-being measures (ps <.001). Social Resources X Group interactions showed associations between high quality social resources and emotional well-being to be stronger for participants with AD relative to CN-Young on nine measures including anger (b=–0.51(0.17), p=.002), physical aggression (b=–0.26(0.11, p=.023), life satisfaction (b=0.35(0.16), p=.033), meaning and purpose (b=0.34,SE=.17,p=.043), positive affect (b=0.36(0.15), p=.018), and perceived stress (b=–0.43(0.17), p=.013). Findings underscore the importance of social resources in older age, particularly among adults with AD. Results suggest that individuals with AD draw upon social resources to improve their emotional well-being, perhaps helping alleviate some of the emotional and behavioral disturbances associated with AD.

